# Childhood conduct problems and adolescent medical service use: serial mediating effects of peer victimization and internalizing problems

**DOI:** 10.1017/S0033291724002241

**Published:** 2024-11

**Authors:** Olivia Crescenzi, Alexa Martin-Storey, Martine Poirier, Stéphanie Boutin, Annie Lemieux, Michèle Déry, Eric Latimer, Caroline E. Temcheff

**Affiliations:** 1 Department of Educational and Counselling Psychology, McGill University, Montreal, QC, Canada; 2 Département de psychoeducation, Université de Sherbrooke, Sherbrooke, QC, Canada; 3 Département des sciences de l’éducation, Université du Québec à Rimouski, Rimouski, QC, Canada; 4 Département de psychologie, Université du Québec à Montréal, Montreal, QC, Canada; 5 Douglas Mental Health University Institute and McGill University, Montreal, QC, Canada

**Keywords:** conduct problems, developmental psychology, internalizing symptoms, medical service use, medicine, peer victimization, psychiatric service use, service use, youth

## Abstract

**Background:**

Adolescents with a history of conduct problems (CP) are at heightened risk of increased service utilization as they develop. While the mechanisms underlying this association are unclear, early CP have also been linked with peer victimization and internalizing problems. The goals of the current study were: (1) to examine peer victimization and internalizing problems as potential serial mediators explaining increased medical and psychiatric service use in adolescents with a history of childhood CP, and; (2) to explore whether the proposed mediation models vary by sex.

**Methods:**

Participants (*N* = 744; 53% boys, Mage = 8.39 years) from an ongoing longitudinal study that began in 2008 in Québec, Canada were recruited and assessed for CP, service use, and other behaviours via self-, parent- and teacher-reported questionnaires. Serial mediation analyses were conducted to examine the effects of peer victimization and internalizing problems on the association between childhood CP and adolescent medical and psychiatric service use, controlling for sex and household income.

**Results:**

Adolescents with childhood CP reported higher medical and psychiatric service use than non-CP peers. Peer victimization and internalizing problems significantly mediated this association in both general medical and psychiatric service use models. The models did not vary by sex.

**Conclusions:**

Findings support higher levels of service use in adolescents with a history of CP, mediated by peer victimization and internalizing problems. Specifically, results highlight the importance of examining peer and socioemotional factors that may explain the increased service usage observed among youth with CP, to support better health outcomes.

## Introduction

**Figure 1.**
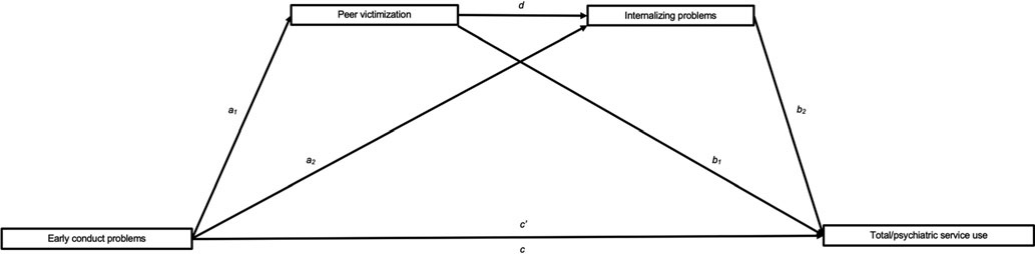
Proposed serial mediational model for total and psychiatric service use. *Note*: The serial mediating effect of peer victimization and internalizing problems in the association between early CP and service use: *a*_n_ is effect of early CP on mediators; *b*_n_ is effect of mediators on service use; *c*′ is direct effect of early CP on service use; *c* is total effect of early CP on service use; *d* is effect of peer victimization on internalizing problems.

Conduct problems (CP), exhibited by up to 4.9% of children, are patterns of behaviour characterized by a spectrum of externalizing and acting-out behaviours (American Psychiatric Association, 2022; Vasileva, Graf, Reinelt, Petermann, & Petermann, 2021). Notably, these behaviours are associated with increased physical and mental health service use across adolescence (Okado, Ewing, Rowley, & Jones, 2017; Temcheff, Martin-Storey, Lemieux, Latimer, & Déry, 2023) and adulthood (Bevilacqua, Hale, Barker, & Viner, 2018; Temcheff et al., 2011a, 2011b; Wertz et al., 2018). Understanding the pathways linking early CP to greater service use is important for supporting the health development of youth with CP. Two inter-related factors that are consequences of early CP and risk factors for service usage are peer victimization (Boutin et al., 2020; Kumpulainen, Räsänen, & Puura, 2001) and internalizing problems (Déry et al., 2017; Herrenkohl et al., 2010). To better understand the processes by which CP leads to service usage, this study tested a model in which peer victimization and internalizing problems acted as mechanisms explaining why adolescents with a history of early CP (i.e. before age 10) use more services than those without history of CP (Fig. 1). The current study offers new insight into the processes through which early CP leads to higher service usage among adolescents and provides a better understanding of potential pathways toward positive health outcomes.

### Theoretical framework

The Life Course Health Development framework (LCHD; Halfon & Hochstein, 2002) provides a framework for examining how early CP, peer victimization, and internalizing problems can lead to increased medical service use in adolescence, and highlights the importance of behavioural factors in addressing health outcomes. In other words, the LCHD framework can explain why youth with CP have worse health outcomes and seek more services than youth without CP. While there are likely many interacting factors that predict service use patterns in youth, existing literature underscores the importance of childhood CP, peer victimization and internalizing problems as critical variables to investigate in this pathway (Kumpulainen et al., 2001; Okado et al., 2017; Rivenbark et al., 2018); this literature will be outlined below.

Previous longitudinal studies have linked childhood CP with increased service use in adulthood. Herrenkohl et al. (2010) found that youth with CP were more likely to use self-reported services at age 27 than those without CP (Herrenkohl et al., 2010). Another study found that early CP led to using higher medical services at age 38 (Rivenbark et al., 2018). Temcheff et al. (2011a, 2011b) employed administrative data of service use, and results indicated that adults with a history of CP had a higher rate of medical visits than those without. While the link between childhood CP and adult service usage has been extensively explored, fewer studies have examined this link in adolescence, an important transitional period that can be highly malleable to interventions (Halfon & Hochstein, 2002). At least three studies have documented the link between childhood CP and increased service use during adolescence (Ford, Hamilton, Meltzer, & Goodman, 2007; Okado et al., 2017; Temcheff et al., 2023). In a longitudinal study, early CP predicted greater use of parent-reported mental health services in adolescence (Okado et al., 2017). Ford et al. (2007) assessed parent-reported service use of children with CP and found that they were more likely to use mental health services in adolescence than non-CP peers (Ford et al., 2007). However, parent reports can reflect recall bias and have shown low-moderate concordance with administrative data (Duncan, Georgiades, Wang, Edwards, & Comeau, 2022). To address this, Temcheff et al. (2023) used administrative records to measure service use trajectories among adolescents with a history of CP. They found that early CP predict increased and chronic service use until age 16 (Temcheff et al., 2023). This literature suggests that childhood CP lead to higher service use in adolescence but, to our knowledge, previous research has yet to investigate psychosocial mechanisms that explain this increased usage.

Previous research demonstrates that early peer victimization may result in increased medical service use and poorer health outcomes (Kumpulainen et al., 2001; Takizawa, Maughan, & Arseneault, 2014). Youth experiencing peer victimization have reported poorer health and higher psychological distress in later life, including depressive and anxious disorders (Sourander et al., 2009; Takizawa et al., 2014). Previous studies have demonstrated that this increased vulnerability to negative health outcomes may be due to maladaptive stress responses caused by early peer victimization (Ouellet-Morin et al., 2013; Takizawa et al., 2014). As supported by the LCHD framework, peer victimization in adolescence is especially distressing due to the importance of peer relations during this period, which can lead to poorer health outcomes and thus higher service usage (Halfon & Hochstein, 2002).

Along with peer victimization, childhood internalizing problems predict higher medical service use in adolescence (Okado et al., 2017) and adulthood (Herrenkohl et al., 2010). One explanation is the comorbidity between internalizing symptoms and somatic complaints (Weersing, Rozenman, Maher-Bridge, & Campo, 2012), as previous literature suggests that individuals with internalizing problems seek medical services for somatic complaints such as abdominal distress (Tylee & Gandhi, 2005). Another explanation is the visibility of these difficulties in home environments or school settings, and the increased likelihood that youth with internalizing symptoms will be referred to specialized mental health services (Cheung, Dewa, Cairney, Veldhuizen, & Schaffer, 2009). These findings may reflect how adolescents experiencing internalizing symptoms are vulnerable to poorer health outcomes, which may lead to higher medical and psychiatric service use (Halfon & Hochstein, 2002). Thus, while the pathway to increased service use is likely complex, existing evidence suggests that early CP, peer victimization, and internalizing problems are all associated with poorer health and subsequent increased service use, and are important variables in this pathway that are specifically worth investigating.

### Conduct problems, peer victimization, and internalizing problems

Previous literature suggests the relevance of exploring peer victimization and internalizing problems as pathways through which CP lead to negative outcomes, including poorer health and medical service use. Children exhibiting CP are more likely to experience adversity in social relationships (e.g. victimization) which can result in internalizing problems (Patterson & Capaldi, 1990), likely via CP augmenting the risk of poor social integration with peers by increasing conflicts, rejection, and subsequent victimization (Patterson & Capaldi, 1990; van Lier et al., 2012). Existing literature supports the link between victimization and CP. Lebrun-Harris, Sherman, Limber, Miller, and Edgerton (2019) reported that youth with CP face a 41% increased risk of being victimized compared to non-CP peers (Lebrun-Harris et al., 2019). CP and peer victimization have also been prospectively linked (Boutin et al., 2020; Boyes, Bowes, Cluver, Ward, & Badcock, 2014).

Previous research has demonstrated that peer victimization, particularly in late childhood and adolescence, can have persistent, adverse effects including negative physical and psychological symptoms (Cubillo, 2022; Takizawa et al., 2014). Findings from several studies support the idea that peer victimization precedes the development of internalizing problems. A systematic review of 28 studies found that peer victimization is a significant risk factor for later depression (Ttofi, Farrington, Lösel, & Loeber, 2011). Additionally, one longitudinal study observed that peer victimization in middle childhood predicted internalizing problems in adolescence and increases in internalizing problems over time (Schwartz, Lansford, Dodge, Pettit, & Bates, 2015). Thus, previous literature supports the interpersonal risk model and highlights the importance of the association between peer victimization and internalizing problems in the health development of youth. Furthermore, existing studies have demonstrated that early CP are associated with a higher likelihood of peer victimization and, as a result, greater internalizing problems (Boutin et al., 2020; Gooren, van Lier, Stegge, Terwogt, & Koot, 2011; van Lier & Koot, 2010). In summary, existing literature suggests that early CP, peer victimization, and internalizing problems are all associated with service usage, and also provides support for examining a sequential pathway wherein CP heightens the risk for peer victimization, leading to increased internalizing symptoms.

### Sex differences

Investigating sex differences in the associations between early CP, peer victimization, internalizing problems, and service usage is important for determining whether service use differs between adolescent boys and girls, therefore supporting the development of sex-specific interventions. Most studies examining sex differences in health service use across CP have observed no effect of sex for service use in adolescence (Okado et al., 2017) or adulthood (Herrenkohl et al., 2010; Rivenbark et al., 2018; Temcheff et al., 2011a, 2011b). However, after examining developmental trajectories of service use, Temcheff and colleagues found that medical service use and initial psychiatric visits were greater among boys with CP when compared to their female counterparts (Temcheff et al., 2023). Regarding the association between internalizing problems and service use, sex differences have not been previously observed (Herrenkohl et al., 2010; Okado et al., 2017). In examining the effect of peer victimization on service use, however, Sourander et al. (2009) found that victimized girls had higher psychiatric visits than non-victimized girls, an effect not seen in boys (Sourander et al., 2009). Given these mixed findings, examining sex differences in the associations between CP, peer victimization, internalizing problems, and service use is warranted.

### Current study

Using longitudinal, administrative health data from a study of youth in Quebec oversampled for CP, the present study addressed two research questions. First, *do peer victimization and internalizing problems mediate the association between childhood CP and adolescent health service utilization*? We hypothesized that peer victimization and internalizing problems would mediate the link between early CP and adolescent medical service use (both for total service use, and for psychiatric visits alone). Since existing literature has demonstrated that childhood CP increases the risk for peer difficulties, internalizing problems, and negative health outcomes in adolescence (Gooren et al., 2011; Lebrun-Harris et al., 2019; Temcheff et al., 2023), we examined CP before age 10 and the outcomes between 11–15 years old, to target the developmental impact of early CP on these outcomes. We chose medical service use as the outcome as children with CP have been shown to be heavy and costly users of medical services in later life (Okado et al., 2017; Temcheff et al., 2011a, 2011b), and we suggest that this higher frequency of service use may reflect poorer physical and mental health as they develop. Our second research question was: *does the proposed mediation model vary by sex*? Given the mixed evidence of sex differences in the focal constructs of the current study, we offer no specific hypothesis as to the direction of sex differences.

## Methods

### Participants

Participants were 744 children with (*n* = 434; 44.2% girls) and without (*n* = 310; 50.3% girls) CP between the ages of 6–9.9 years old at study inception (Time 1: *M* = 8.39 years, s.d. = 0.93). Participants were recruited for a longitudinal study conducted in four regions in Québec, Canada, from 155 French-speaking schools across eight public school boards from 2008–2010. Nearly 60% of children attended a primary school with a high deprivation index. Schools' indices of deprivation are calculated by the Ministry of Education based on the national census (Ministère de l’Éducation et de l'Enseignement Supérieur, 2021). The deprivation index includes the proportion of families (a) where the mother does not hold a diploma, (b) where parents are unemployed, and (c) who are near or below the low-income threshold. Schools receive a score on a scale from 1–10, with scores between 7 and 10 indicating high deprivation levels (Ministère de l’Éducation et de l'Enseignement Supérieur, 2021). Two recruitment strategies were employed. Most children with CP (*n* = 339) were identified and recruited on the basis of their having received psychosocial services for these problems at their schools. In order to recruit equal numbers of girls and boys, and considering the fact that more boys were receiving services for CP, all girls under the age of 10, and one out of four boys were randomly selected to participate (participation rate = 75.1%). Following selection, 339 children were confirmed as having clinical levels of CP, as assessed by scores within the clinical range (*t* score ≥ 70) of the DSM-oriented scales for either conduct problems or oppositional defiant problems of the Achenbach System of Empirically Based Assessments (ASEBA; Achenbach & Rescorla, 2001), according to parent- or teacher-report. This method is both specific and sensitive in identifying CP (Lapalme, Bégin, Le Corff, & Déry, 2020). The other participants were identified by screening 881 children not receiving psychosocial interventions in schools located in disadvantaged neighborhoods, using the same scales (participation rate = 71.5%), which resulted in identifying 95 additional children with clinical levels of CP. Children in the control group (*n* = 310) were randomly selected from recruits who did not meet criteria for conduct problems or oppositional defiant problems.

### Procedure

**Figure 2.**
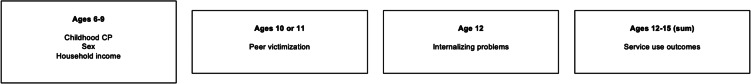
Measures assessed at each age of study.

Following ethics approval, consent was provided by participants' primary caregivers, which also included parents' permission for research assistants to contact their child's teacher. Children provided assent. Graduate-level research assistants visited the family homes every 12 months and administered questionnaires to parents and children, and teachers were phone-interviewed. The measures taken at each age are presented in Fig. 2 for clarity.

### Measures

#### Conduct problems

The DSM-oriented scales for conduct problems and oppositional/defiant problems of the Child Behavioural Checklist (CBCL; 6–18 years old) and the Teacher Report Form (TRF) of the ASEBA (Achenbach & Rescorla, 2001) were used. Both scales are consistent with respective diagnostic criteria for conduct disorder or oppositional defiant disorder outlined in the *Diagnostic and Statistical Manual of Mental Disorders* (DSM-IV, American Psychiatric Association, 1994). These criteria remain consistent in the DSM-5-TR (APA, 2022). The Conduct Problems scale comprised 17 items for parents (Time 1-Time 4; *α* = 0.86–0.87) and 13 items for teachers (Time 1-Time 4; *α* = 0.91–0.93). The Oppositional/Defiant Problems scale included 5 items for parents (Time 1-Time 4; *α* = 0.83–0.85) and 5 items for teachers (Time 1-Time 4; *α* = 0.90–0.92). Responses for both scales are scored as a 3-point Likert scale ranging from 0 (not true) to 2 (very true), with higher scores indicating higher levels of CP severity. A child was considered to have CP (binary variable) if either parents or teachers reported CP in the clinical range (*t* score ≥ 70) before age 10. This method of determining clinical status was used to optimize the selection of children with clinical CP in the sample, based on the data available from both parents and teachers (Lapalme et al., 2020). In addition, ratings for the CP group (*n* = 376) were as follows: 177 participants assigned to CP due to *both* parent- and teacher-rated clinical CP, 138 participants assigned to CP due to *parent*-rated clinical CP (but not teacher), and 61 participants assigned to CP due to *teacher-*rated clinical CP (but not parent). Moreover, moderate to high correlations were found between parent- and teacher-reports on the Conduct Problems scale (*r* = 0.60**) and the Oppositional Defiant Problems scale (*r* = 0.56**). χ^2^ analyses examining sex differences in the Conduct Problems and Oppositional Defiant Problems scales are provided in supplementary material.

#### Peer victimization

To assess peer victimization, children completed a modified version of the self-report victimization scale from the *Étude longitudinale du développement des enfants du Québec* (Institut de la statistique du Québec, 2005) from Time 2 to Time 5, and analyses were conducted using the score reported at age 10 or 11, depending which of the two ages the children were during these timepoints. The questionnaire included 8 items asking whether the child had experienced different types of bullying (verbal, physical, relational) and was scored on an ordinal scale including 0 (never), 1 (1–2 times), or 2 (more often than 2 times), with higher scores indicating more instances of peer victimization. Examples of questions included ‘Has anyone at school ever called you names or said something mean to you?’ and ‘Has anyone at school ever pushed, hit, or kicked you?’. The omega alpha (appropriate for ordinal data; Gadermann, Guhn, & Zumbo, 2019) for Time 2-Time 5 of this scale ranged from 0.76 to 0.78, which was deemed adequate (Rouhi Safaei et al., 2022).

#### Internalizing problems

Internalizing problems were assessed using the Youth Self-Report questionnaire of the ASEBA (Achenbach & Rescorla, 2001) when participants were age 12. The scale includes 42 items assessing symptoms of anxiety (‘I am nervous or tense’), depression (‘I am unhappy, sad, or depressed’), social withdrawal (‘I would rather be alone than with others’), and somatic complains (‘Aches or pains’). Items were rated on a 3-point scale from 0 (not true) to 2 (very true or often true). Raw scores were transformed to t-scores, and higher t-scores indicated higher levels of internalizing problems. The internal consistency for this scale ranged from 0.88 to 0.90.

#### Medical service utilization

Medical service utilization data were retrieved from the *Régie de l'assurance maladie du Québec* (RAMQ), which is the single-payer public health plan in Québec. The total number of annual and psychiatric visits between participants' 12th and 15th birthdays were used. *Total medical service visits* were obtained by summing all yearly medical and psychiatric visits. *Psychiatric visits* included visits to psychiatrists and other physicians that ultimately led to a psychiatric diagnosis or treatment for a psychiatric condition.

#### Control variables

The present study controlled for the effects of sex (male or female at birth, reported by parents) and household income at study inception. Household income was reported by primary caregivers and was based on an ordinal scale adapted from the Quebec Child Mental Health Survey (Valla et al., 1997). The median household income fell between $50 000 to $59 000.

### Statistical analyses

Preliminary analyses were conducted using IBM SPSS Statistics 27. Frequencies and descriptives were calculated for all variables (Table 1). Descriptives of the service use outcomes indicated high asymmetry (skewness > 3.0) and leptokurtic distributions (kurtosis > 10.0) for both outcomes. Thus, values for both outcomes were truncated at the 98th percentile (Kline, 2008). Given that both outcomes (total and psychiatric visits) were initially count variables, square root transformations were applied to transform them into continuous variables (Tabachnick & Fidell, 1996). Next, bivariate correlations among study variables were computed. From the original sample of 744, the data for 30 participants were removed due to values missing on both the outcome and mediator variables. Frequencies run on the predictors indicated that the household income variable was missing for 8 participants, so these were also removed. Analyses were conducted with a final sample size of 706 participants.

**Table 1.** Descriptive statistics of key variables

Variable	*M* (s.d.)	Median	%
CP status			
CP			50.5
Non-CP			49.5
Sex			
Male			53.2
CP			(51.3)
Non-CP			(48.7)
Female			46.8
CP			(49.7)
Non-CP			(50.3)
Household income		$50 000–$59 000	
Peer victimization			
Never			19.5
CP			(12.6)
Non-CP			(26.6)
1–2 times			27.8
CP			(21.6)
Non-CP			(34.1)
More often than 2 times			52.7
CP			(65.8)
Non-CP			(39.3)
Internalizing problems (*t-*score)	48.52 (10.54)		
CP	50.88 (10.57)		
Non-CP	46.04 (9.93)		
Total medical service use visits	17.80 (23.05)		
CP	21.30 (28.92)		
Non-CP	14.45 (14.83)		
Psychiatric service use visits	1.47 (4.50)		
CP	2.57 (5.96)		
Non-CP	0.42 (1.88)		

*Note*: *N* = 706.

**Figure 3.**
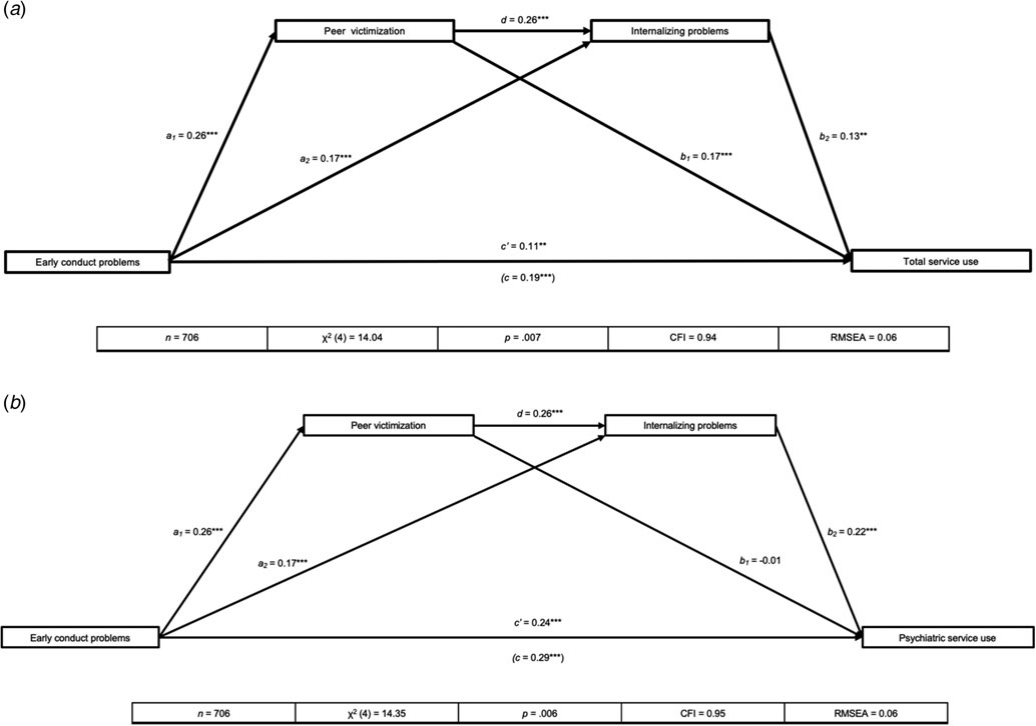
(a) Serial mediational model for total service use (controlling for sex and household income at T1). (b) Serial mediational model for psychiatric service use (controlling for sex and household income at T1). *Note*: The serial mediating effect of peer victimization on internalizing problems in the association between early CP and total service use. *p < 0.05, **p < 0.01, ***p < 0.001. All presented effects are standardized; *a*_n_ is effect of early CP on mediators; *b*_n_ is effect of mediators on total service use; *c*′ is direct effect of early CP on total service use; *c* is total effect of early CP on total service use; *d* is effect of peer victimization on internalizing problems. *Note*: The serial mediating effect of peer victimization on internalizing problems in the association between early CP and psychiatric service use. *p < 0.05, **p < 0.01, ***p < 0.001. All presented effects are standardized; *a*_n_ is effect of early CP on mediators; *b*_n_ is effect of mediators on psychiatric service use; *c*′ is direct effect of early CP on psychiatric service use; *c* is total effect of early CP on psychiatric service use; *d* is effect of peer victimization on internalizing problems.

Next, serial mediational models were conducted using MPlus Version 8.7 (Muthén & Muthén, 2017). Structural equation modelling was used to evaluate the effects of peer victimization (ages 10 or 11; mediator 1) and internalizing problems (age 12; mediator 2) in the association between childhood CP (before age 10, predictor) and adolescent medical service use outcomes (ages 12–15; total service use first, psychiatric visits alone second), while controlling for sex and household income on the health service use outcomes. Maximum likelihood with robust standard error (MLR) estimation followed by bootstrapping were used to test the models, with one thousand bootstrap samples used to calculate the 95% confidence intervals of the indirect effects. Model fit was examined using the χ^2^ statistic, Comparative Fit Index (CFI), and Root Mean Square Error of Approximation (RMSEA), using the following cut-offs: CFI at 0.90 and below 0.08 for RMSEA (Vandenberg & Lance, 2000). Standardized estimates were reported for each step within the path models (Fig. 3a, b), and 95% bias-corrected confidence intervals were examined for significance of indirect effects (Preacher & Hayes, 2008). Finally, sex differences for both models were evaluated using multi-group analyses by comparing the difference in χ^2^ statistic between a sex-stratified baseline model with no equality constraints, against a model with equality constraints imposed.

## Results

Prior to conducting path model analyses, bivariate correlations were computed (Table 2). The predictor variable and mediators were statistically significantly correlated with the two service use outcome variables and each other. Baseline associations between CP and parent- and teacher-reported internalizing problems showed that children with CP had higher levels of internalizing problems than children without CP, which is presented in the supplementary material.

**Table 2.** Bivariate correlations of key variables

Variable	1	2	3	4	5	6	7
1. Conduct problems	1.00	−0.015	−0.307**	0.258**	0.230**	0.149**	0.239**
2. Sex	–	1.00	−0.103**	−0.017	−0.096*	0.045	0.012
3. Household income	–	–	1.00	−0.095*	−0.175**	−0.075	−0.084
4. Peer victimization	–	–	–	1.00	0.302**	0.181**	0.089*
5. Internalizing problems	–	–	–	–	1.00	0.181**	0.190**
6. Total service use	–	–	–	–	–	1.00	0.617**
7. Psychiatric service use	–	–	–	–	–	–	1.00

*Note*: **p < 0.01, *p < 0.05, *n* = 706.

### Path model analyses

#### Model 1: total service use

The fit of the serial mediational model (*n* = 706) for total service use was adequate (see Fig. 3a). While the χ^2^ statistic was significant, findings were still considered robust due to the large sample size (Hu & Bentler, 1999). Results showed that early CP, peer victimization, and internalizing problems all had statistically significant direct links with total medical service use from 12–15 years old. As well, early CP were associated with a higher likelihood of experiencing peer victimization, and higher peer victimization was subsequently associated with higher levels of internalizing problems, which sequentially was associated with higher total medical service use by the 12–15 age range.

In total, three mediation pathways were tested in the serial mediation model. More specifically, the indirect effect of peer victimization on the association between early CP and total service use was statistically significant (*B* = 0.05, 95% CI 0.09–0.31). As well, the indirect effect of internalizing problems on the association between early CP and total service use was statistically significant (*B* = 0.02, 95% CI 0.03–0.21). Lastly, the serial mediating effect between peer victimization and internalizing problems on the association between early CP and total service use was also statistically significant (*B* = 0.01, 95% CI 0.01–0.08). Results supported a serial mediating model between early CP and total medical service use via peer victimization and subsequent internalizing. This association remained significant when accounting for ADHD symptoms (see supplementary materials). For further information regarding these associations with both total and psychiatric service use while accounting for attentional symptoms and adolescent conduct problems, please see supplementary material.

Furthermore, we conducted supplementary mediation analyses with a third outcome (total service use with psychiatric visits removed). This model is included in supplementary material, and suggests that while the direct effect of CP on service use was no longer significant, the indirect effect of peer victimization and internalizing problems was still significant.

#### Model 2: psychiatric service use

As with Model 1, the fit of the serial mediational model (*n* = 706) for psychiatric service use was adequate (see Fig. 3b). Results showed that early CP and internalizing problems both had statistically significant direct links to psychiatric service use from 12–15 years old. However, peer victimization did not have a statistically significant direct link with psychiatric service use. As was the case with general medical service usage, early CP were associated with a higher likelihood of experiencing peer victimization and a higher level of subsequent internalizing problems. Higher peer victimization was also associated with higher levels of internalizing problems, which were then sequentially associated with higher psychiatric service use.

As in Model 1 above, three indirect pathways were tested. More specifically, the indirect effect of peer victimization on the association between early CP and psychiatric service use was not statistically significant (*B* = −0.001, 95% CI −0.06 to 0.04). In contrast, the indirect effect of internalizing problems on the association between early CP and psychiatric service use was statistically significant (*B* = 0.04, 95% CI 0.04–0.16). Lastly, the serial mediating effect between peer victimization and internalizing problems on the association between early CP and psychiatric service use was also statistically significant (*B* = 0.02, 95% CI 0.02–0.06). Results supported a serial mediating model where peer victimization and internalizing problems mediated the association between early CP and psychiatric service use. Again, these associations remained significant while accounting for ADHD symptoms and adolescent CP, as are presented in the supplementary materials.

### Invariance by sex

In order to test invariance by sex for both models, baseline models with no equality constraints imposed were compared against fixed models with equality constraints on regression coefficients. Analyses for both models indicated that the χ^2^ difference was not statistically significant, suggesting that the models were statistically similar for boys and girls.

## Discussion

Youth with CP are more likely than those without these problems to have higher medical and psychiatric service use in adolescence (Okado et al., 2017; Temcheff et al., 2023) and adulthood (Rivenbark et al., 2018; Temcheff et al., 2011a, 2011b). The current study extends previous literature by using a longitudinal approach relying on administrative health data to examine how peer victimization and internalizing problems mediate this link. Findings supported the first hypothesis such that early CP was linked with increased experiences of peer victimization, which in turn were linked with increased internalizing problems and subsequent medical and psychiatric service use in adolescence. Findings also indicated that the proposed model did not differ for boys and girls.

Consistent with existing theoretical frameworks on adolescent health outcomes (Halfon & Hochstein, 2002; Patterson & Capaldi, 1990), these findings supported a model wherein peer victimization and internalizing problems in early adolescence operated sequentially, with regards to the link between childhood CP and service usage. These findings indicate the importance of examining psychosocial factors that explain the increased need for health service usage among youth with CP, to begin understanding pathways to support better health outcomes. As found in previous work, youth with greater levels of CP experienced more peer victimization (Boutin et al., 2020; Boyes et al., 2014; Lebrun-Harris et al., 2019) and internalizing symptoms (Martin-Storey et al., 2018; Van der Ende, Verhulst, & Tiemeier, 2016; Wertz et al., 2015), and peer victimization was linked with subsequent internalizing problems (Card & Hodges, 2008; van Lier et al., 2012). The current findings extend this literature, moreover, by suggesting the sequential nature of these links. This pathway may suggest that in order to receive support for depressive and anxious symptoms, some youth with CP seek out medical and psychiatric services at a higher frequency than their peers without CP.

Interestingly, although peer victimization was significantly correlated with psychiatric visits, these concepts were not directly linked in the path model. One possible explanation for this finding is that when internalizing problems emerged as a result of peer victimization, participants with CP used more psychiatric services or were more often referred by parents, teachers, or counselors to these services (Cheung et al., 2009). These findings nevertheless support the serial nature of peer victimization and subsequent internalizing problems that increase psychiatric visits among many adolescents with CP.

Regarding the second research question, sex invariance was found for both models, indicating that the serial effect of peer victimization and internalizing problems on increased adolescent medical service use is similar for boys and girls with CP. The absence of significant sex differences is consistent with previous findings that did not identify significant sex differences for service use in adolescence (Okado et al., 2017) or adulthood (Herrenkohl et al., 2010; Rivenbark et al., 2018; Temcheff et al., 2011a, 2011b), but contradicts other studies that suggest that sex differences may exist when developmental trajectories are examined (Temcheff et al., 2023). Temcheff et al. (2023) observed sex differences in total medical services and psychiatric visits. However, this difference was found using a trajectory and not a static measure of service use like the one employed in the current study. It is possible that sex differences were simply not captured by the use of a static measure, and that sex differences in this population may present over time, and not within the timeframe of the current study. These findings suggest that a history of CP and peer victimization are important factors for physicians to consider when assessing a child presenting for internalizing symptoms, regardless of their sex.

### Limitations

The current study has several strengths. By utilizing administrative health data from medical records in order to evaluate service use, the risk of social desirability and recall bias often reflected in self- or parent-reports (Duncan et al., 2022) was minimized. Moreover, the longitudinal nature of the study allowed for the temporal ordering of variables and thus provided new insight about the serial effects of peer victimization and internalizing problems in adolescence as a result of childhood CP.

Certain limitations should also be considered when interpreting the current findings. First, recruitment for the study over-sampled for children with CP as rated by parents and teachers. While this represented a strength in that it allowed for the evaluation of youth with more severe behavioural difficulties, it limits the generalizability of findings to the broader youth population. In addition, these parent- and teacher-reports were not systematically verified by clinicians on the study team. However, utilizing both parent- and teacher-reports has been shown to be a sensitive measure for detecting childhood CP (Lapalme et al., 2020). Moreover, all children were recruited in Québec, which has a particular culture as well as educational and health care system. These findings would need to be replicated in other countries or regions. Finally, while existing literature supports the association between peer victimization and subsequent internalizing problems in adolescents (Patterson & Capaldi, 1990; Schwartz et al., 2015; Ttofi et al., 2011), it is important to note that the link between these two processes is dynamic and bidirectional (Reijntjes, Kamphuis, Prinzie, & Telch, 2010).

## Conclusions

In the current study, experiences of peer victimization and higher internalizing problems serially mediated the association between childhood CP and increased adolescent medical and psychiatric service use. Higher use of services due to increased access to care in youth with CP may reflect that youth with these problems are accessing needed services. However, higher service use may also reflect poorer health status for some youth in this population and is potentially cause for concern. Ultimately, these findings underscore the importance of future research aimed at identifying and evaluating other psychosocial factors that may increase the likelihood of children with CP using more services than peers in adolescence, to promote better health outcomes. The current study suggests that youth with CP are more likely to be victimized by peers and develop internalizing problems, and are subsequently more likely to seek medical and psychiatric services. These findings offer new insight into why children with CP use increased medical services in adolescence and support the hypothesis that some youth with CP rely on medical and psychiatric services as they develop, possibly due to higher risk for psychosocial difficulties. These findings also have relevant clinical implications; they suggest the importance of sensitizing clinicians and practitioners in schools to screening for this type of sequential process that culminates in increased use of services. Specifically, assistance in reducing the amount of peer victimization toward children with CP, and also secondary prevention of internalizing problems among vulnerable youth with multiple risk factors (CP and being victimized by peers) should be prioritized.

## Supporting information

Crescenzi et al. supplementary material Crescenzi et al. supplementary material

## Supplementary material

The supplementary material for this article can be found at https://doi.org/10.1017/S0033291724002241.
