# Supplementary material for: Childhood conduct problems and adolescent medical service use: serial mediating effects of peer victimization and internalizing problems
Source: Psychol Med. 2024 Dec 2;54(15):4193–202. doi: 10.1017/S0033291724002241 (PMC11650198; doi:10.1017/S0033291724002241)
Supplement: Crescenzi et al. supplementary material [file S0033291724002241sup001.docx]

**Supplementary material 1 - Table 1S**

*Chi-square analyses by sex*

| Scale/Timepoint | Χ^2^ | df | *p* |
| --- | --- | --- | --- |
| Conduct/T1 | .211 | 1 | .65 |
| Oppositional/T1 | .714 | 1 | .40 |
| Conduct/T2 | .166 | 1 | .68 |
| Oppositional/T2 | .444 | 1 | .51 |
| Conduct/T3 | 1.24 | 1 | .27 |
| Oppositional/T3 | .226 | 1 | .63 |
| Conduct/T4 | .000 | 1 | .99 |
| Oppositional/T4 | .662 | 1 | .42 |

*Note*. *n* = 744.

**Supplementary material 2**

The baseline association between CP and internalizing problems (CBCL and TRF; (Achenbach & Rescorla, 2001)) was analyzed. Youth with CP (*M* = 62.21, *SD* = 9.51) had significantly higher parent-reported internalizing problems than youth without CP at baseline (*M* = 56.26, *SD* = 10.47), *t*(730.60) = 8.10, *p* < .001. Similarly, youth with CP (*M* = 62.33, *SD* = 9.18) had significantly higher teacher-reported internalizing problems than youth without CP at baseline (*M* = 55.38, *SD* = 9.61), *t*(704) = 9.83, *p* < .001.

**Supplementary material 3 - Figure 1S**

*Serial mediational model for total service use (minus psychiatric visits)*


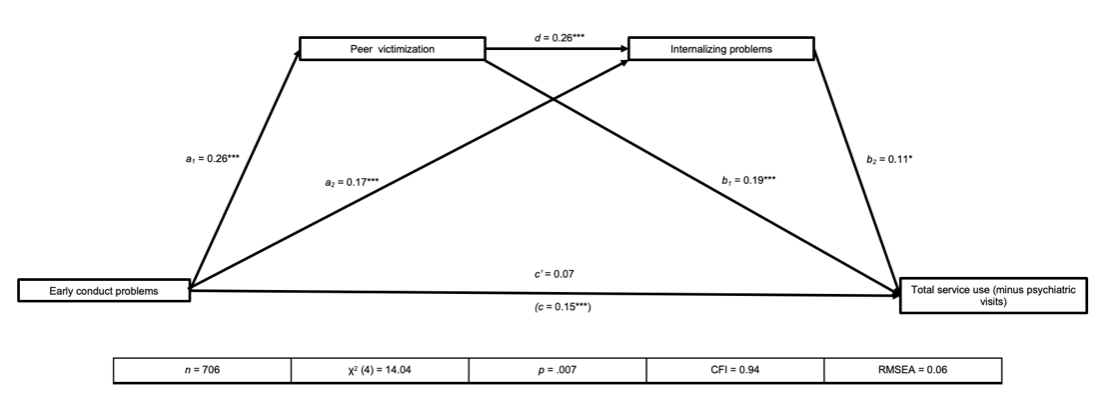


*Note.* **p* < .05, ***p* < .01, ****p* < .001.

**Supplementary material 4**

Extant literature has shown that up to 26% of children with CP have comorbid ADHD (Mohammadi et al., 2021). Given that ADHD has also been associated with higher medical and psychiatric service use (Du Rietz et al., 2020), we wanted to demonstrate that the effect of CP persists, even when controlling for ADHD. We used a composite measure of ADHD at T1 that was constructed in a similar manner to the T1 measure of CP (dichotomized score; 0 = No ADHD diagnosis by parent and teacher, 1 = ADHD diagnosis by parent and/or teacher). ADHD symptomatology was measured using the parent- and teacher-reported Conners Rating Scales (Conners, Sitarenios, Parker, & Epstein, 1998b, 1998a). While ADHD was correlated with both CP (*r* = .38, *p* < .001) and service use (total service use *r* = .18, *p* < .001; psychiatric service use *r* = .18, *p* < .001), regression analyses revealed that, while T1 ADHD was related to both service use outcomes, CP remained pertinent and still predicted both outcomes while accounting for ADHD (see Tables 2S and 3S below).

**Table 2S**

*Regression results predicting total service use with ADHD*

|  | *B* | β | *p* |
| --- | --- | --- | --- |
| Conduct problems | 4.27 | .093 | .042 |
| ADHD | 8.46 | .148 | .001 |
| *R^2^* |  |  | .041** |

*Note*. *B* indicates unstandardized coefficients. β indicates standardized (beta) coefficients. *p < .05. **p < .01.

**Table 3S**

*Regression results predicting psychiatric service use with ADHD*

|  | *B* | β | *p* |
| --- | --- | --- | --- |
| Conduct problems | 1.81 | .201 | < .001 |
| ADHD | 1.10 | .099 | .029 |
| *R^2^* |  |  | .065** |

*Note*. *B* indicates unstandardized coefficients. β indicates standardized (beta) coefficients. *p < .05. **p < .01.

**Supplementary material 5**

Supplementary analyses were conducted by adding adolescent conduct problems (CP at age 12; YSR, (Achenbach & Rescorla, 2001)) to the mediation models at the same step as internalizing problems for both service use outcomes. Results indicated that adolescent CP was not directly related to either service use outcome, nor was the indirect effect of either model with adolescent CP included significant once the effect of childhood CP was accounted for. However, in both cases, the indirect effect of the models without adolescent CP remained significant. These models are presented in Figure 1S and Figure 2S below.

**Figure 2S**

*Serial mediational model for total service use with adolescent CP added*


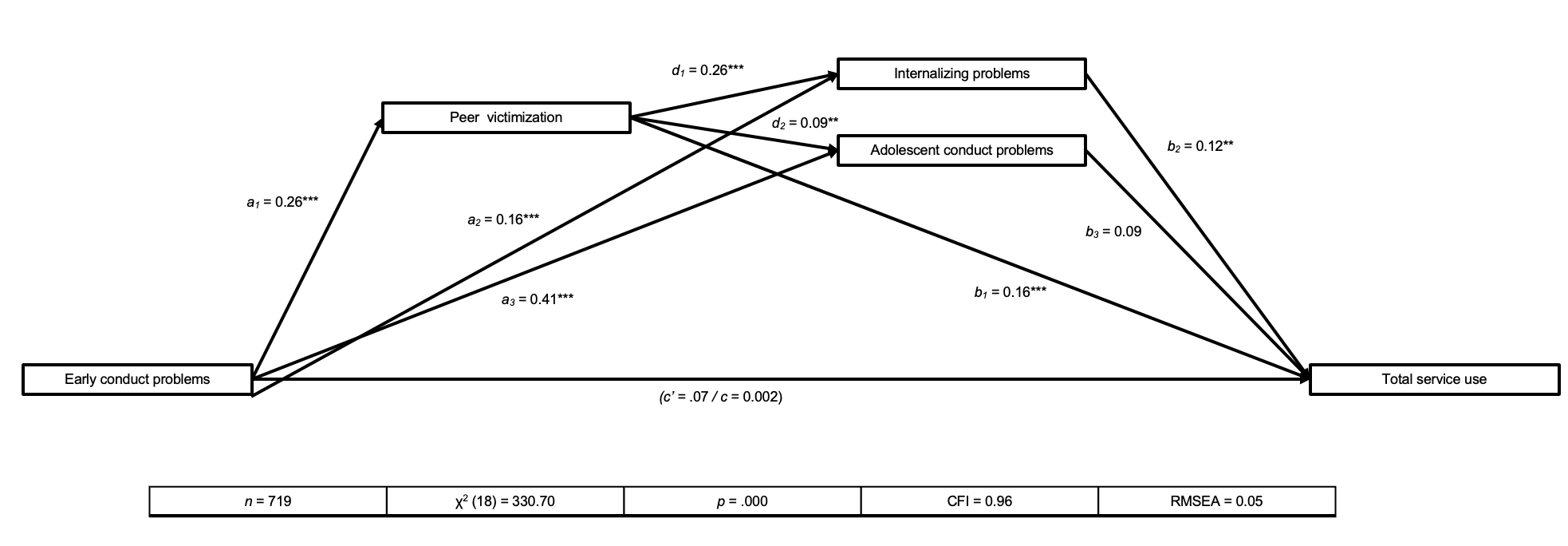


*Note.* **p* < .05, ***p* < .01, ****p* < .001.

**Figure 3S**

*Serial mediational model for psychiatric service use with adolescent CP added*


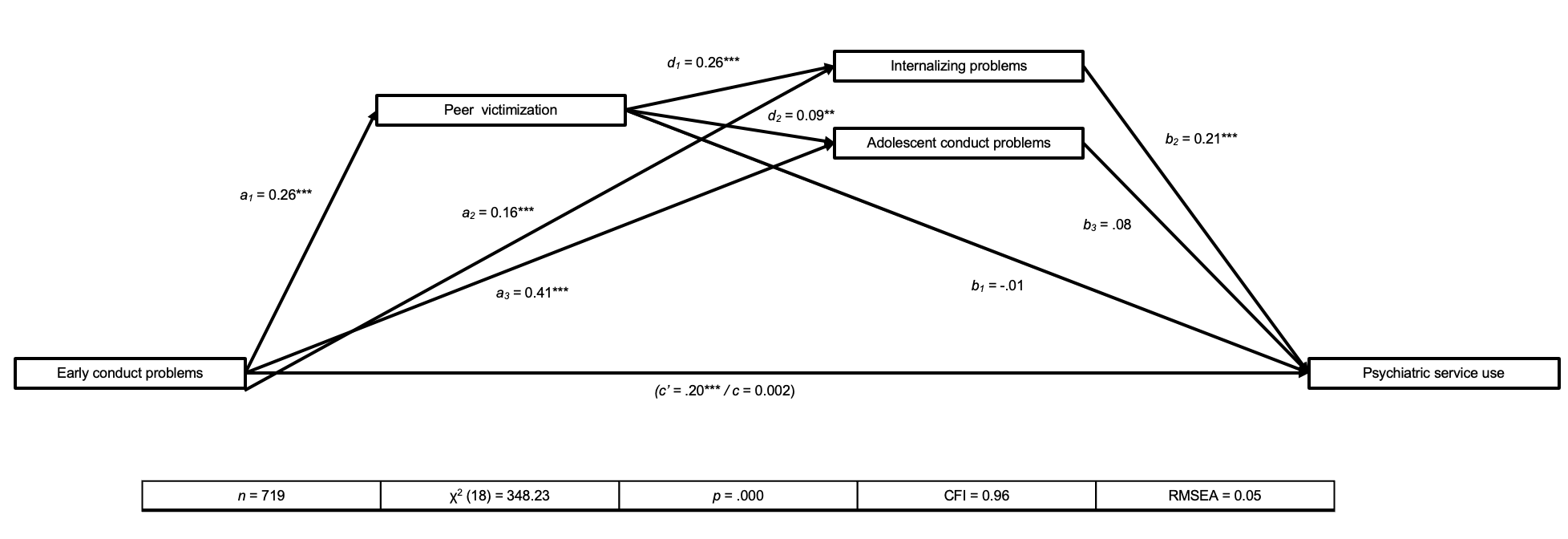


*Note.* **p* < .05, ***p* < .01, ****p* < .001.

**Supplementary material – References**

Achenbach, T. M., & Rescorla, L. A. (2001). *Manual for the ASEBA school‐age forms & profiles*. Research Center for Children, Youth, & Families: University of Vermont.

Conners, C. K., Sitarenios, G., Parker, J. D. A., & Epstein, J. N. (1998a). Revision and Restandardization of the Conners Teacher Rating Scale (CTRS-R): Factor Structure, Reliability, and Criterion Validity. *Journal of Abnormal Child Psychology*, *26*(4), 279–291. https://doi.org/10.1023/A:1022606501530

Conners, C. K., Sitarenios, G., Parker, J. D. A., & Epstein, J. N. (1998b). The Revised Conners’ Parent Rating Scale (CPRS-R): Factor Structure, Reliability, and Criterion Validity. *Journal of Abnormal Child Psychology*, *26*(4), 257–268. https://doi.org/10.1023/A:1022602400621

Du Rietz, E., Jangmo, A., Kuja-Halkola, R., Chang, Z., D’Onofrio, B. M., Ahnemark, E., … Larsson, H. (2020). Trajectories of healthcare utilization and costs of psychiatric and somatic multimorbidity in adults with childhood ADHD: A prospective register-based study. *Journal of Child Psychology and Psychiatry*, *61*(9), 959–968. https://doi.org/10.1111/jcpp.13206

Mohammadi, M.-R., Zarafshan, H., Khaleghi, A., Ahmadi, N., Hooshyari, Z., Mostafavi, S.-A., … Salmanian, M. (2021). Prevalence of ADHD and Its Comorbidities in a Population-Based Sample. *Journal of Attention Disorders*, *25*(8), 1058–1067. https://doi.org/10.1177/1087054719886372
